# A Tablet-Based Assessment of Rhythmic Ability

**DOI:** 10.3389/fpsyg.2019.02471

**Published:** 2019-11-01

**Authors:** Theodore P. Zanto, Namita T. Padgaonkar, Alex Nourishad, Adam Gazzaley

**Affiliations:** ^1^Department of Neurology, University of California, San Francisco, San Francisco, CA, United States; ^2^Neuroscape, University of California, San Francisco, San Francisco, CA, United States; ^3^Interdepartmental Neuroscience Program, University of California, Los Angeles, Los Angeles, CA, United States; ^4^Department of Psychiatry, Mount Sinai Beth Israel, New York, NY, United States; ^5^Department of Physiology and Department of Psychiatry, University of California, San Francisco, San Francisco, CA, United States

**Keywords:** sensorimotor synchronization, rhythm, multimodal integration, mobile tablet, aging, musicianship

## Abstract

The exponential rise in use of mobile consumer electronics has presented a great potential for research to be conducted remotely, with participants numbering several orders of magnitude greater than a typical research paradigm. Here, we attempt to demonstrate the validity and reliability of using a consumer game-engine to create software presented on a mobile tablet to assess sensorimotor synchronization, a proxy of rhythmic ability. Our goal was to ascertain whether previously observed research results can be replicated, rather than assess whether a mobile tablet achieves comparable performance to a desktop computer. To achieve this, younger (aged 18–35 years) and older (aged 60–80 years) adult musicians and non-musicians were recruited to play a custom-designed sensorimotor synchronization assessment on a mobile tablet in a controlled laboratory environment. To assess reliability, participants performed the assessment twice, separated by a week, and an intra-class correlation coefficient (ICC) was calculated. Results supported the validity of this approach to assessing rhythmic abilities by replicating previously observed results. Specifically, musicians performed better than non-musicians, and younger adults performed better than older adults. Participants also performed best when the tempo was in the range of previously-identified preferred tempos, when the stimuli included both audio and visual information, and when synchronizing on-beat compared to off-beat or continuation (self-paced) synchronization. Additionally, high ICC values (>0.75) suggested excellent test–retest reliability. Together, these results support the notion that consumer electronics running software built with a game engine may serve as a valuable resource for remote, mobile-based data collection of rhythmic abilities.

## Introduction

In recent years, mobile technology has advanced to the level where it has become possible to easily collect data from people remotely for research purposes, to assess things such as blood flow, heart rate variability, respiratory rate, gait statistics, cognitive function, and psychological state to name a few (Harrison et al., 2011; Mishra et al., 2016; Michard, 2017; Munster-Segev et al., 2017; Rye Hanton et al., 2017). Despite this uptick in remote data collection, real concerns exist regarding the validity and reliability of remote data collection devices and software. Aside from the uncontrolled environment where participants reside, multiple sources of variability may stem from the technology itself, such as different interfaces (e.g., phone, tablet, computer), different operating systems, and different software used to collect data. At a more fundamental level, mobile consumer devices are not necessarily designed to simultaneously produce stimuli and acquire performance data with the precise timing required by typical research projects (Ng and Dietz, 2014; Ritter et al., 2015; Arsintescu et al., 2017; Begel et al., 2017).

As a step toward validating the use of mobile devices for research purposes in this domain, we used popular game engine software to create an assessment of sensorimotor synchronization, a proxy of rhythmic ability. The overall goal was to replicate previously identified findings, but to do so with a mobile tablet in a controlled laboratory environment. As such, our goal was not to ascertain whether a mobile tablet achieves comparable performance to a desktop computer. Rather, replicating previously established findings using a paradigm that demands high temporal precision will provide important evidence toward the validity of tablet-based data collection to assess rhythmic ability. This goal is in-line with our previous validation of using tablets to assess spatial attention (Rolle et al., 2015, 2017), except the current paradigm places greater demands on the tablet’s timing/processing ability in terms of stimulus presentation and data collection, which occur simultaneously and continuously.

The sensorimotor synchronization assessment characterizes the ability to tap in-phase with a metronome (i.e., on-beat), anti-phase with a metronome (i.e., 180° off-beat), or continue the metronome tempo after it stops. In addition to these three tasks, three tempos (350, 525, 750 ms inter-onset-interval) are assessed and three stimulus types are used to present the metronome (visual-only, auditory-only, audio-visual). Research assessing rhythmic ability typically uses asynchrony (the temporal difference between where a tap occurred and where it should have occurred) and the variance (or standard deviation) of the asynchrony (for reviews, see Repp, 2005c; Repp and Su, 2013). Using these metrics, previous research have shown in-phase sensorimotor synchronization yields greater performance than tasks that require anti-phase synchronization (Yamanishi et al., 1980; Kelso, 1981; Mechsner et al., 2001; Spencer and Ivry, 2007) or continuing a metronome tempo after it stops (Semjen et al., 2000; Flach, 2005; Snyder et al., 2006; Ito et al., 2013). Additionally, adult sensorimotor synchronization performance data has indicated a preferred tempo between 400 and 700 ms inter-onset-interval (Fraisse, 1982; Moelants, 2002; McAuley et al., 2006), with lowered performance on faster and slower tempos (Parncutt, 1994; McAuley et al., 2006; Delevoye-Turrell et al., 2014; Zamm et al., 2018). Finally, prior research has suggested that stimuli presented via multiple modalities simultaneously result in benefits to rhythmic performance (Elliott et al., 2010; Wing et al., 2010). Taken together, it is hypothesized that tablet-based rhythmic performance will be greatest during in-phase synchronization tasks, for medium tempos (i.e., 525 ms inter-onset-interval), and when stimuli are presented bimodally (i.e., audio-visual).

In addition to detailed characterizations of these different influences on sensorimotor synchronization, previous research has also indicated that rhythmic ability is greater in musicians compared to non-musicians (Pressing and Jolley-Rogers, 1997; Repp, 2010), and that rhythmic ability declines in advanced age (Thaut et al., 1997; Krampe et al., 2005; Bangert and Balota, 2012; Iannarilli et al., 2013; Thompson et al., 2015). In an attempt to replicate these findings, younger adults (aged 18–35 years) and older adults (aged 60–77 years) were recruited for this study. Within each age group, musicians (>10 years formal training) and non-musicians (<5 years formal training) were recruited. In line with prior research, it was hypothesized that musicians would yield greater synchronization performance than non-musicians, whereas younger adults would exhibit greater performance than older adults.

To assess reliability of the data, participants engaged in the same sensorimotor synchronization assessment twice, separated by a week, and an intra-class correlation coefficient (ICC) was calculated. Test–retest reliability was characterized as excellent (ICC > 0.75), good (ICC = 0.6–0.74), fair (ICC = 0.4–0.59), or poor (ICC < 0.4) (Cicchetti, 1994). Together, replicating previous results and demonstrating good-to-excellent ICC, would provide evidence that this sensorimotor synchronization assessment as presented on a mobile tablet is a valid and reliable method to characterize rhythmic ability. Furthermore, this will provide more evidence supporting the utility of mobile tablets more broadly, by using consumer game engines to create software to collect data for research purposes (Rolle et al., 2015, 2017).

## Materials and Methods

### Participants

Nineteen younger adult non-musicians (mean age = 22.8 years, *SD* = 4.6 years), 22 younger adult musicians (mean age = 20.3 years, *SD* = 1.7 years), 14 older adult non-musicians (mean age = 68.4 years, *SD* = 3.8 years), and 16 older adult musicians (mean age = 68.1 years; *SD* = 4.7 years) gave informed consent to participate in the study according to procedures approved by the Committee for Human Research at the University of California, San Francisco. All participants were screened to ensure that they were in normal health, had no history of neurological, psychiatric, or vascular disease, were not depressed, and were not taking any psychotropic or hypertensive medications. All participants had normal or corrected to normal vision and hearing. Musicians were identified as having 10 or more years of experience with: musical instrument, singing, and/or dancing; non-musicians were identified as having 5 or less years of such experience.

### Neuropsychological Testing

Participants in the older age group were administered 10 neuropsychological tests of executive and memory function, and were found to be cognitively intact (within 2 SD) relative to normative values from age-matched controls. Neuropsychological testing was performed on a separate day from the two behavioral assessment days and included the following tests: Mini Mental State Examination (Folstein et al., 1975), geriatric depression scale (Yesavage et al., 1982), visual-spatial function (modified Rey-Osterrieth), visual-episodic memory (memory for details of a modified Rey-Osterrieth Complex Figure), visual-motor sequencing (trail making test B), Logical Memory I, Verbal Paired Associates I, and Visual Reproduction II [all from the Weschler Memory Scale Revised (Wechsler, 1987)], the California Verbal Learning Test (Delis et al., 2000), Stroop interference (Stroop, 1935), and WAIS digit symbol test (Wechsler, 2008).

### Experimental Design

Two separate behavioral assessments were administered, one for sensorimotor synchronization ability and one for multiple other cognitive functions. All participants performed both the cognitive battery and sensorimotor synchronization assessment on their 1^st^ visit, and then repeated only the sensorimotor synchronization assessment on their 2^nd^ visit exactly 1 week later.

**FIGURE 1:**
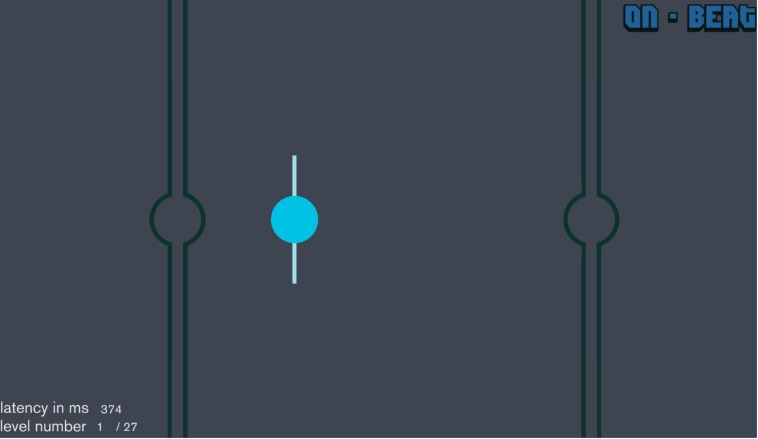
Screenshot of experimental paradigm.

The sensorimotor synchronization assessment was programed in Unity, executed on a Microsoft Surface Pro 3 and designed to assess rhythmic capabilities as measured by the ability to tap different metronome-like sequences (Figure 1). The assessment measured rhythmic ability across 27 levels, which consisted of parametrically manipulating three variables: tempo of the metronome, the audio-visual information provided, and the rhythmic task performed. The tempo varied between slow (750 ms), medium (525 ms), and fast (350 ms) inter-onset intervals (IOIs). The stimuli presented varied between a visual-only stimulus where the movement of a ball between two lines on each side of the screen denoted the metronome “beat,” an audio-only stimulus where a distinct tone denoted the metronome “beat,” and an audio-visual stimulus where these cues were integrated. Lastly, participants were asked to perform three tasks: (1) On-beat: tap along with each stimulus event (i.e., beat: sound onset and/or when the ball touched the lines at either side of the screen), (2) Off-beat: tap half-way between each stimulus event, or (3) Continuation: after four stimulus events (i.e., four beats), the stimuli were discontinued and participants had to continue the metronomic rhythm by tapping for four beats without disrupting the tempo. After the four-beat “silent period” where participants were to tap, stimuli were resumed for another four beats and followed by another four-beat “silent period” where participants were instructed to tap. The stimuli and silent periods continued to alternate for the duration of the level. Together, the sensorimotor synchronization consists of 27 levels (3 tempos × 3 stimulus types × 3 tasks) each lasting approximately 30 s. Therefore, participants underwent approximately 13.5 min of time-on-task.

### Materials

Visual stimuli consisted of a blue circle with a small vertical line through it moving horizontally from one side of the screen to the other, passing between larger vertical lines, a pair on the left side of the screen and a pair on the right – each equidistant from the center of the screen – such that when the circle was in the middle of the vertical lines it changed direction, indicating the beat (Figure 1). Auditory stimuli consisted of a 50 ms long 800 Hz tone. Sound intensity was set to a comfortable listening level. After each level, participants were provided with their average absolute offset (asynchrony) in ms to provide feedback on their performance and were then provided instructions for the next level to complete. During gameplay, task instructions remained in the upper right corner of the screen (i.e., “on-beat,” “off-beat,” or “continuation”). Moreover, the lower left corner of the screen indicated which level the participant was on and a measure of tap offset was displayed to provide online feedback (Figure 1). Additional feedback was provided in the form of a vertical dashed line to indicate when the screen was touched and was located where the visual ball was at the time of tap onset, thereby providing a visualization of the tap offset. This dashed line was not present during the auditory-only conditions.

A cognitive battery was administered using the Adaptive Cognitive Evaluation (ACE) platform presented on an iPad (see Anguera et al., 2016 for details). Tests evaluating the following cognitive abilities were used: basic response time, multi-tasking, response inhibition, sustained attention/impulse control, task switching, visual search, visuo-spatial working memory. Task order within ACE was randomized between participants. Participants were given either ACE or the sensorimotor synchronization assessment as their first task randomly as well. Results for the ACE battery will not be discussed but generally were consistent with the results from the neuropsychological testing.

### Data Acquisition and Analyses

All participants were seen at either the University of California, San Francisco or University of California, Berkeley campuses. The same tablet was used in both locations and not different tablets of the same model. Participants were given scripted, verbal instructions for both the cognitive battery and the rhythm assessment. Each of the rhythm assessment levels were first explained and performed by the experimenter, and then the participants were instructed to practice each task for a minimum of 30 s. Once participants were comfortable with all three tasks, the assessment began. At the beginning of each level, participants were given 3 s of “get ready” time before they were able to start synchronizing. After 3 s, the visual “get ready” cue disappeared and data was recorded beginning with the participant’s first tap on the screen. As such, participants were able to see/hear the metronome for a minimum of 3 s until they were ready to begin. Unfortunately, the number of beats to start tapping was not recorded and so we were unable to assess this metric, although participants generally began immediately after the 3 s get ready period.

Accuracy was determined by calculating the percentage of correct taps. For each stimulus event (i.e., where a participant should have tapped), a window was defined as ± IOI/2. Within that window, an incorrect response would be marked if no tap occurred or if more than one tap occurred. As such, accuracy measures the ability to tap 1:1 with the stimuli. All other performance metrics were calculated from “correct” taps. Asynchrony was calculated as the absolute offset in milliseconds from the instructed tap onset. Standard deviations were calculated from tap offsets. A composite *rhythm score* was calculated by z-scoring the accuracy, asynchrony, and standard deviation separately across all participants and assessment levels, and then averaging the three z-scores together per participant and level. Of note, asynchrony and standard deviation z-scores were multiplied by −1 prior to averaging together to ensure that higher values relate to better performance. The utility of the rhythm score lies in the fact that accuracy, standard deviation, and asynchrony all capture different aspects of synchronization performance. While it could be argued that some of these component metrics are more important than others, a weighted average was not conducted because the relative importance of each of these metrics is unknown and likely subjective. Additionally, relative phase and vector length were calculated using the CircStat toolbox in Matlab (Berens, 2009) by converting tap offsets to radians.

Accuracy, asynchrony, standard deviation, vector length and the composite rhythm score were all subjected to a repeated measures analysis of variance (ANOVA) with Age (younger, older) and Experience (musician, non-musician) as between subject factors and Task (on-beat, off-beat, continuation), Stimulus (audio-visual, audio-only, visual-only), and Tempo (slow, medium, fast) as within subject factors. A Greenhouse–Geisser correction was applied as necessary. Main effects and interactions were interrogated via *t*-tests. A Bonferroni correction was applied for all *post hoc t*-tests. Differences in relative phase were assessed via a Watson–Williams test and Bonferroni corrected.

Test–retest reliability was assessed via the ICC (Shrout and Fleiss, 1979). The ICC model used was ICC (Mishra et al., 2016; Michard, 2017), as defined by Shrout and Fleiss (1979), in which both the performance data and participants are treated as random effects to assess reliability at a single point in time. This form of ICC utilizes a two-way ANOVA to estimate the correlation of performance between sessions. Test–retest reliability was characterized as excellent (ICC > 0.75), good (ICC = 0.6–0.74), fair (ICC = 0.4–0.59), or poor (ICC < 0.4) (Cicchetti, 1994). Five participants did not complete the second experimental visit and so these participants were not included in the analysis (1 young non-musician, 1 older non-musician, and 3 older musicians).

## Results

As noted above, it was hypothesized that synchronization performance would be greatest in musicians (vs. non-musicians), young adults (vs. older adults), during the on-beat task (vs. continuation or off-beat), with audio-visual stimuli (vs. audio or video only), and during the medium tempo (vs. fast or slow tempos). Synchronization performance was assessed via relative phase, vector length, accuracy, (absolute) asynchrony, standard deviation and a composite rhythm score. Rather than conducting a series of one-way ANOVAs for each of the five hypotheses (with the exception of relative phase), data were submitted to a five-way ANOVA, and main effects were assessed.

### Assessment of Experience

**FIGURE 2:**
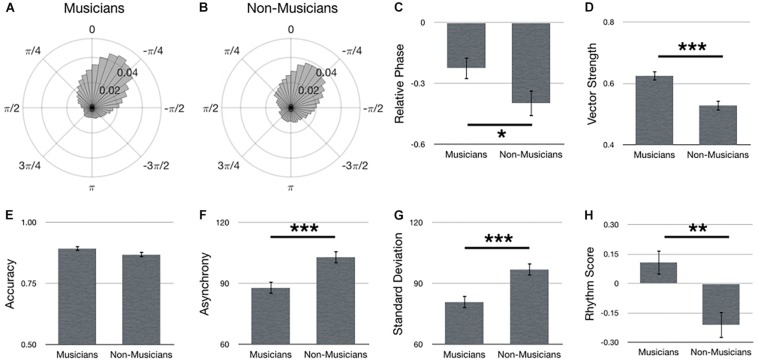
Main effects for Experience. Polar histograms of all taps (normalized as probability) are depicted for **(A)** musicians and **(B)** non-musicians. The zero point at top indicates where participants should have tapped, while positive values indicate late taps and negative values are early. Comparisons between groups are depicted for **(C)** relative phase, **(D)** vector length, **(E)** accuracy, **(F)** absolute asynchrony, **(G)** standard deviation, and **(H)** rhythm score. Error bars indicate standard error of the mean. ^∗^*p* < 0.05, ^∗∗^*p* < 0.01, ^∗∗∗^*p* < 0.001.

Figure 2 summarizes main effects for Experience. Polar histograms of all the taps in relative phase shows that taps generally precede stimulus onset, regardless of musical expertise (Figures 2A,B). Importantly, as hypothesized, musicians outperformed non-musicians in terms of a smaller relative phase [Figure 2C; *F*(1,69) = 4.20, *p* = 0.044], larger vector length [Figure 2D; *F*(1,67) = 18.37, *p* < 0.001, $\eta_{\text{p}}^{2}$ = 0.22], smaller asynchrony [Figure 2F; *F*(1,67) = 16.01, *p* < 0.001, $\eta_{\text{p}}^{2}$ = 0.19], smaller standard deviation [Figure 2G; *F*(1,67) = 16.18, *p* < 0.001, $\eta_{\text{p}}^{2}$ = 0.20] and larger rhythm score [Figure 2H; *F*(1,67) = 12.14, *p* = 0.001, $\eta_{\text{p}}^{2}$ = 0.15]. No difference between musicians and non-musicians was observed for accuracy [Figure 2E; *F*(1,67) = 2.99, *p* = 0.088, $\eta_{\text{p}}^{2}$ = 0.04].

### Assessment of Age

**FIGURE 3:**
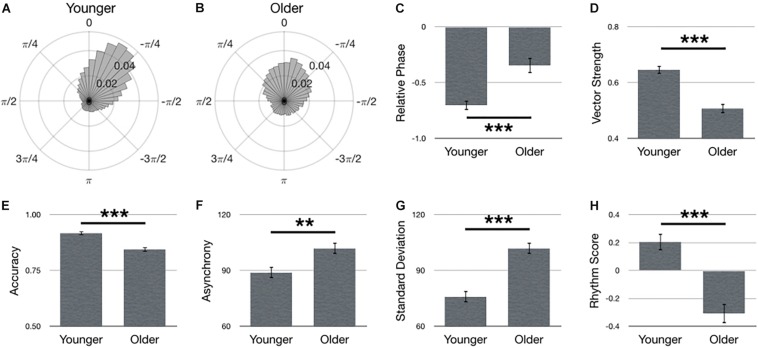
Main effects for Age. Polar histograms of all taps (normalized as probability) are depicted for **(A)** younger and **(B)** older adults. The zero point at top indicates where participants should have tapped, while positive values indicate late taps and negative values are early. Comparisons between groups are depicted for **(C)** relative phase, **(D)** vector length, **(E)** accuracy, **(F)** absolute asynchrony, **(G)** standard deviation, and **(H)** rhythm score. Error bars indicate standard error of the mean. ^∗^*p* < 0.05, ^∗∗^*p* < 0.01, ^∗∗∗^*p* < 0.001.

Figure 3 summarizes main effects for Age. Polar histograms of all the taps in relative phase shows that taps generally precede stimulus onset, regardless of age group, although older adults produced more late taps (Figures 3A,B). Contrary to our hypothesis, relative phase was smaller in the older adult group compared to younger adults [Figure 3C; *F*(1,69) = 20.77, *p* < 0.001]. However, this effect was due to the quantity of late taps in the older adult group, as well as the increased variance overall, as depicted by a lower vector length in older, compared to younger, adults [Figure 3D; *F*(1,67) = 37.28, *p* < 0.001, $\eta_{\text{p}}^{2}$ = 0.36]. Moreover, as hypothesized, younger adults outperformed older adults in terms of larger accuracy [Figure 3E; *F*(1,67) = 24.96, *p* < 0.001, $\eta_{\text{p}}^{2}$ = 0.27], smaller asynchrony [Figure 3F; *F*(1,67) = 11.21, *p* = 0.001, $\eta_{\text{p}}^{2}$ = 0.14], smaller standard deviation [Figure 3G; *F*(1,67) = 47.74, *p* < 0.001, $\eta_{\text{p}}^{2}$ = 0.42] and larger rhythm score [Figure 3H; *F*(1,67) = 31.49, *p* < 0.001, $\eta_{\text{p}}^{2}$ = 0.32].

### Assessment of Task

**FIGURE 4:**
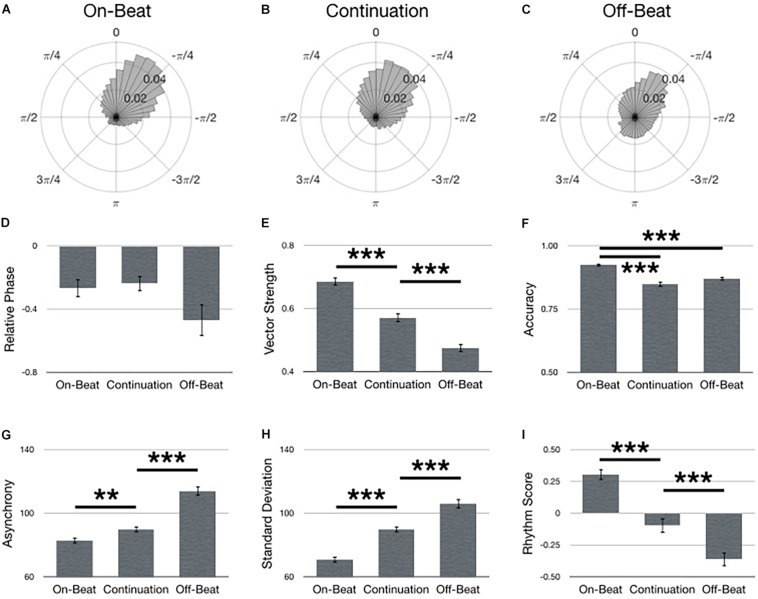
Main effects for Task. Polar histograms of all taps (normalized as probability) are depicted for **(A)** on-beat, **(B)** continuation, and **(C)** off-beat tasks. The zero point at top indicates where participants should have tapped, while positive values indicate late taps and negative values are early. Comparisons between tasks are depicted for **(D)** relative phase, **(E)** vector length, **(F)** accuracy, **(G)** absolute asynchrony, **(H)** standard deviation, and **(I)** rhythm score. Error bars indicate standard error of the mean. ^∗^*p* < 0.05, ^∗∗^*p* < 0.01, ^∗∗∗^*p* < 0.001.

Figure 4 summarizes main effects for Task. Polar histograms of all the taps in relative phase shows that taps generally precede stimulus onset, regardless of task type (Figures 4A–C). As hypothesized, the on-beat task yielded the best synchronization performance as indicated by a larger vector length [Figure 4E; *F*(2,134) = 142.17, *p* < 0.001, $\eta_{\text{p}}^{2}$ = 0.68], larger accuracy [Figure 4F; *F*(2,134) = 49.96, *p* < 0.001, $\eta_{\text{p}}^{2}$ = 0.43], smaller asynchrony [Figure 4G; *F*(2,134) = 66.50, *p* < 0.001, $\eta_{\text{p}}^{2}$ = 0.50], smaller standard deviation [Figure 4H; *F*(2,134) = 144.72, *p* < 0.001, $\eta_{\text{p}}^{2}$ = 0.68] and larger rhythm score [Figure 4I; *F*(2,134) = 112.80, *p* < 0.001, $\eta_{\text{p}}^{2}$ = 0.63]. No difference was observed between tasks for relative phase [Figure 4D; *F*(2,210) = 1.69, *p* = 0.187].

### Assessment of Stimulus

**FIGURE 5:**
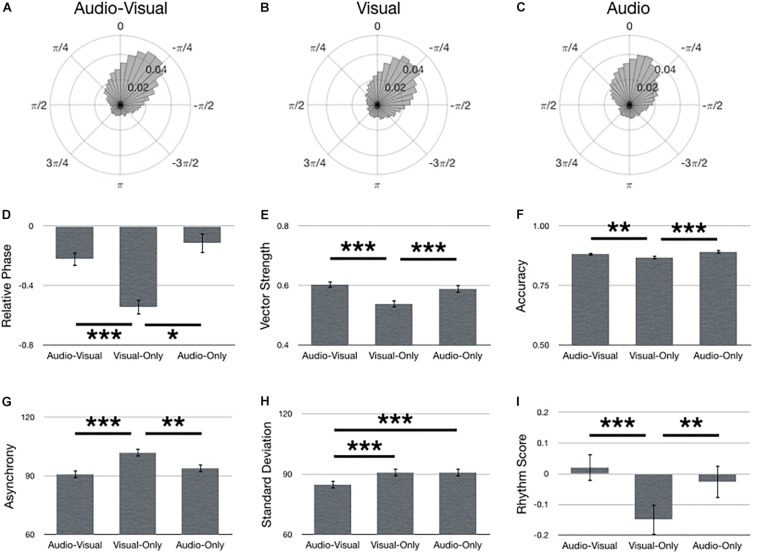
Main effects for Stimulus. Polar histograms of all taps (normalized as probability) are depicted for **(A)** audio-visual, **(B)** visual-only, and **(C)** audio-only. The zero point at top indicates where participants should have tapped, while positive values indicate late taps and negative values are early. Comparisons between stimulus types are depicted for **(D)** relative phase, **(E)** vector length, **(F)** accuracy, **(G)** absolute asynchrony, **(H)** standard deviation, and **(I)** rhythm score. Error bars indicate standard error of the mean. ^∗^*p* < 0.05, ^∗∗^*p* < 0.01, ^∗∗∗^*p* < 0.001.

Figure 5 summarizes main effects for Stimulus. Polar histograms of all the taps in relative phase shows that taps generally precede stimulus onset, regardless of stimulus type (Figures 5A–C). In support of our hypothesis, audio-visual stimuli yielded the lowest standard deviation (i.e., best synchronization performance) [Figure 5H; *F*(2,134) = 8.48, *p* < 0.001, $\eta_{\text{p}}^{2}$ = 0.11]. However, every other metric of synchronization performance only provided partial support for our hypothesis. Specifically, performance with audio-visual stimuli did not significantly differ from audio-only and both stimulus types were better than visual-only in terms of smaller relative phase [Figure 5D; *F*(2,210) = 15.57, *p* < 0.001], larger vector length [Figure 5F; *F*(2,134) = 26.27, *p* < 0.001, $\eta_{\text{p}}^{2}$ = 0.28], larger accuracy [Figure 5F; *F*(2,134) = 12.46, *p* < 0.001, $\eta_{\text{p}}^{2}$ = 0.16], smaller asynchrony [Figure 5G; *F*(2,134) = 19.17, *p* < 0.001, $\eta_{\text{p}}^{2}$ = 0.22], and larger rhythm score [Figure 5I; *F*(2,134) = 15.13, *p* < 0.001, $\eta_{\text{p}}^{2}$ = 0.18]. Although vector length did not show a significant difference between audio-visual and audio-only, it was trending in that direction (*p* = 0.089, uncorrected).

### Assessment of Tempo

**FIGURE 6:**
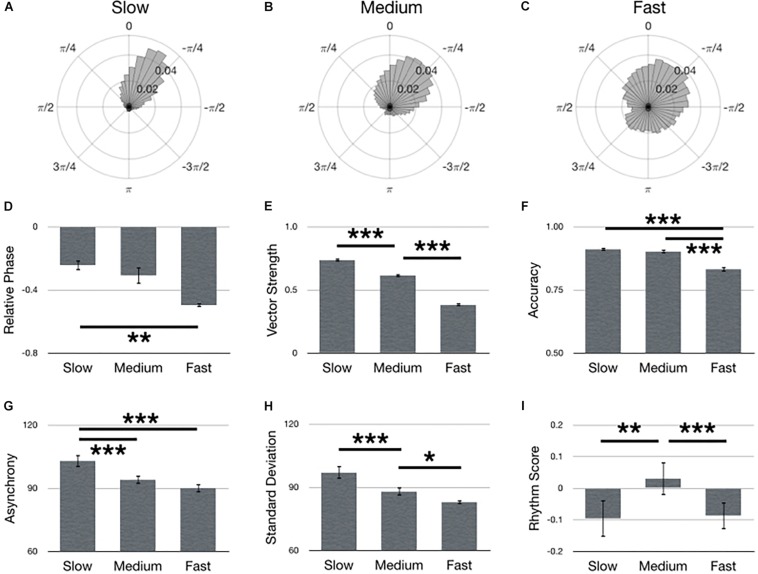
Main effects for Tempo. Polar histograms of all taps (normalized as probability) are depicted for **(A)** slow, **(B)** medium, and **(C)** fast tempos. The zero point at top indicates where participants should have tapped, while positive values indicate late taps and negative values are early. Comparisons between tempos are depicted for **(D)** relative phase, **(E)** vector length, **(F)** accuracy, **(G)** absolute asynchrony, **(H)** standard deviation, and **(I)** rhythm score. Error bars indicate standard error of the mean. ^∗^*p* < 0.05, ^∗∗^*p* < 0.01, ^∗∗∗^*p* < 0.001.

Figure 6 summarizes main effects for Tempo. Polar histograms of all the taps in relative phase shows that taps generally precede stimulus onset, regardless of tempo (Figures 6A–C). In support of our hypothesis, the medium tempo yielded the largest rhythm score (i.e., best synchronization performance) [Figure 6I; *F*(2,134) = 6.34, *p* = 0.002, $\eta_{\text{p}}^{2}$ = 0.09]. However, several metrics provided only partial support for our hypothesis. Specifically, accuracy during the medium tempo was comparable to slow, but better (larger) than the fast tempo [Figure 6F; *F*(2,134) = 73.32, *p* < 0.001, $\eta_{\text{p}}^{2}$ = 0.52], while asynchrony during the medium tempo was comparable to fast, but better (smaller) than the slow tempo [Figure 6G; *F*(2,134) = 19.91, *p* < 0.001, $\eta_{\text{p}}^{2}$ = 0.23]. Furthermore, relative phase during the medium tempo was comparable to slow, but only the slow tempo was significantly better (smaller) than fast [Figure 6D; *F*(2,210) = 8.60, *p* < 0.001]. Contrary to our hypothesis, the slow tempo yielded largest vector length [Figure 6F; *F*(2,134) = 519.33, *p* < 0.001, $\eta_{\text{p}}^{2}$ = 0.89], while the fast tempo exhibited the smallest standard deviation [Figure 6H; *F*(2,134) = 19.46, *p* < 0.001, $\eta_{\text{p}}^{2}$ = 0.23].

### Test–Retest Reliability

**FIGURE 7:**
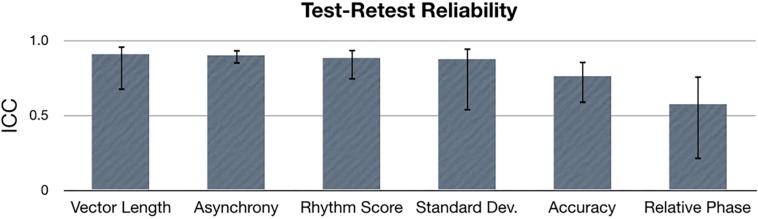
Intraclass correlation (ICC) values for each sensorimotor synchronization metric. Error bars indicate 95% confidence intervals.

To assess test–retest reliability, an ICC was calculated for each synchronization metric: relative phase, vector length, accuracy, asynchrony, standard deviation and rhythm score. Data was averaged over all tasks, stimulus types and tempos, while data from each age and experience group were concatenated for the ICC calculation. Results show that ICC values were greater than 0.75 for vector length, asynchrony, rhythm score, standard deviation and accuracy, indicating excellent test–retest reliability for these metrics (Figure 7). ICC for relative phase suggested fair test–retest reliability (ICC = 0.58).

### Exploratory Analysis

As the results above suggest the data is both valid (via replication) and reliable (via ICC), it is therefore of interest to capitalize on the unique structure of the paradigm to assess previously unexplored relationships between age, musical experience, stimulus, tempo, and task type. Whereas the *a priori* hypotheses were based on main effects, this exploratory analysis will focus on the highest level interactions from the five-way ANOVAs. Specifically, vector length exhibited a four-way interaction between Age × Task × Stimulus × Tempo [*F*(8,536) = 2.15, *p* = 0.041, $\eta_{\text{p}}^{2}$ = 0.03]. Accuracy exhibited three three-way interactions between Experience × Age × Stimulus [*F*(2,134) = 3.75, *p* = 0.029, $\eta_{\text{p}}^{2}$ = 0.05], Age × Task × Tempo [*F*(4,268) = 10.37, *p* < 0.001, $\eta_{\text{p}}^{2}$ = 0.13], and Task × Stimulus × Tempo [*F*(8,536) = 2.73, *p* = 0.010, $\eta_{\text{p}}^{2}$ = 0.04]. Asynchrony exhibited a four-way interaction between Age × Task × Stimulus × Tempo [*F*(8,536) = 4.21, *p* = 0.001, $\eta_{\text{p}}^{2}$ = 0.06]. Standard deviation exhibited two four-way interactions between Experience × Age × Task × Tempo [*F*(4,268) = 3.09, *p* = 0.035, $\eta_{\text{p}}^{2}$ = 0.04] and Age × Task × Stimulus × Tempo [*F*(8,536) = 2.54, *p* = 0.030, $\eta_{\text{p}}^{2}$ = 0.04]. Finally, the rhythm score also showed a four-way interaction between Experience × Age × Task × Tempo [*F*(4,268) = 3.23, *p* = 0.024, $\eta_{\text{p}}^{2}$ = 0.05].

**FIGURE 8:**
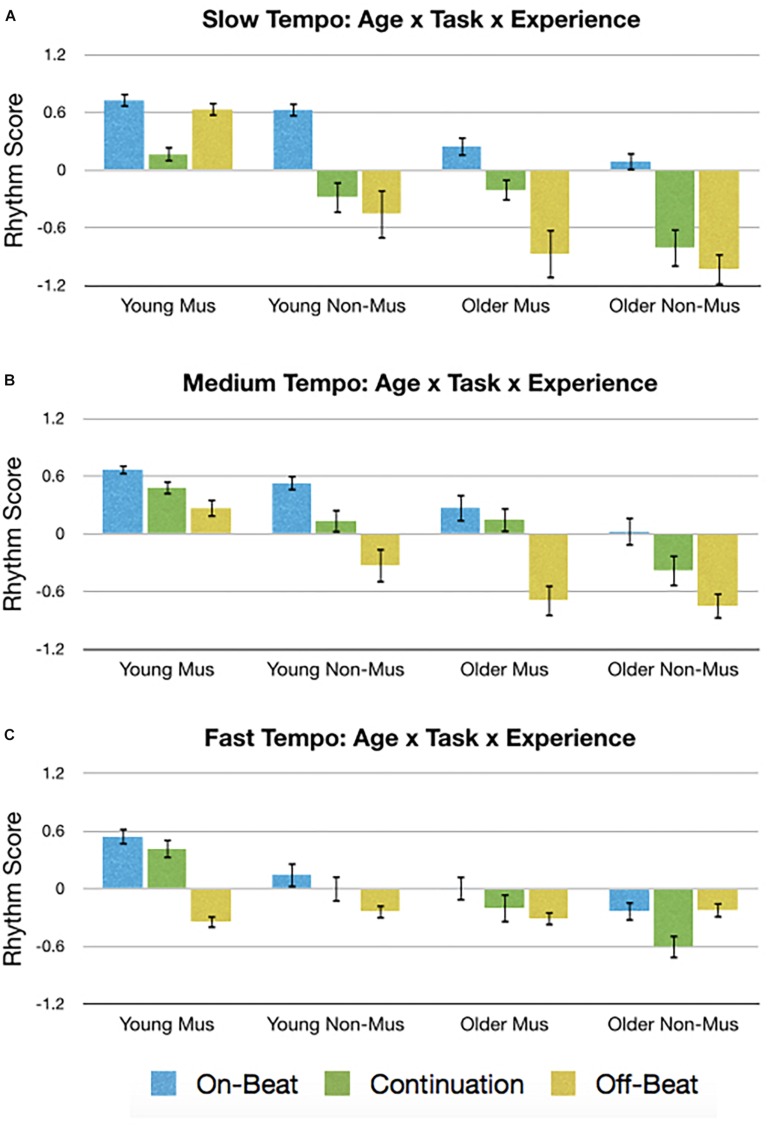
Graphical depiction of the Age × Task × Experience interaction for the **(A)** slow tempo, **(B)** medium tempo, and **(C)** fast tempo. Error bars indicate standard error of the mean. Mus, musicians; Non-Mus, non-musician.

Rather than conducting an exhaustive assessment of all these complex interactions, only the Experience × Age × Task × Tempo interaction from the rhythm score was briefly explored. The rhythm score was

selected for this analysis because it provided the most support across all five of our *a priori* hypotheses (Table 1). The data giving rise to the Experience × Age × Task × Tempo interaction is presented in Figure 8. Through visual inspection, general trends can be seen that gave rise to main effects, such as younger > older adults, musicians > non-musicians, and on-beat > continuation > off-beat. However, two cases are apparent where off-beat > continuation and is comparable to on-beat: young musicians during the slow tempo (Figure 8A) and older non-musicians during the fast tempo (Figure 8C). *Post hoc t*-tests were then conducted to assess these potential contributors to the four-way interaction. Results show that for younger musicians during the slow tempo, off-beat performance was greater (larger rhythm score) than continuation [*t*(21) = 4.34, *p* < 0.004, *d* = 1.32] and not significantly different from on-beat [*t*(21) = 1.65, *p* = 0.460, *d* = 0.27]. Similarly, older non-musicians during the fast tempo exhibited off-beat performance greater than continuation [*t*(13) = 3.09, *p* = 0.036, *d* = 0.99], which did not significantly differ from on-beat [*t*(21) = 0.07, *p* = 1.00, *d* = 0.02].

**TABLE 1:** Summary of synchronization metrics as they pertain to the five hypotheses on experience, age, task, stimulus, and tempo.

	**Experience**	**Age**	**Task**	**Stimulus**	**Tempo**
Relative phase	X			*/*	*/*
Vector length	X	X	X	*/*	
Accuracy		X	X	*/*	*/*
Asynchrony	X	X	X	*/*	*/*
Standard deviation	X	X	X	X	
Rhythm score	X	X	X	*/*	X

*An ‘X’ indicates support for the hypothesis, whereas a ‘*/*’ indicates partial support.*

## Discussion

The current results demonstrate the utility of using a tablet-based platform to assess sensorimotor synchronization abilities as defined by multiple measures of performance: relative phase, vector length, accuracy, asynchrony, standard deviation, and a composite rhythm score. Results generally support previous research and our hypotheses (Table 1), such that musicians exhibit greater synchronization ability than non-musicians, younger adults exhibit greater synchronization ability than older adults, and synchronization ability was greatest during the on-beat task, medium tempo (i.e., 525 ms IOI) and when stimuli contain both audio and visual information. Finally, it was shown that overall performance largely yielded excellent test–retest reliability. Together, these results show that tablet-based mobile platforms can be a valid (via replication) and reliable (via ICC) means to collect measures of sensorimotor synchronization ability.

One of the most well-documented findings in the sensorimotor synchronization literature is that of the negative mean asynchrony, which refers to the tendency for participants to tap ahead of (or early to) the stimulus. This asynchrony typically ranges between 20 and 100 ms prior to stimulus onset (Kolers and Brewster, 1985; Dunlap, 1910; Aschersleben and Prinz, 1995; Thaut et al., 1997; Aschersleben, 2002). Here, we replicate this asynchrony as is apparent from the polar histograms and measures of relative phase in Figures 2–6. However, our measure of asynchrony reports *absolute* asynchrony, rather than using the traditional metric of mean asynchrony, so that values closer to zero would be indicative of better performance, rather than reflecting a sensitivity to late taps or increased variance around zero. The consequence of using absolute values yields larger asynchrony measures, which were observed to be 95 ms when averaged over all groups and experimental factors. Although this falls at the outer edge of previously observed results, when we calculate traditional mean (not absolute) asynchrony, the negative mean asynchrony is 59 ms, thereby placing these results well within previously published ranges.

### Effects of Experience

It was hypothesized that musicians would perform better than non-musicians. Previous research has reported the negative mean asynchrony to be smaller (i.e., closer to no asynchrony) in musicians, compared to non-musicians by 10 to 30 ms (Aschersleben, 1994; Repp and Doggett, 2007; Repp, 2010). Here, the effects of musical experience was not only replicated, but were within the range of previously reported data. Specifically, the asynchrony in musicians were 10 or 15 ms smaller than non-musicians, when calculating asynchrony as a mean or absolute measure, respectively (Figure 2F).

Prior research has also shown a smaller standard deviation in musicians. Using a 500 ms IOI Repp (2010) observed musicians to yield a standard deviation that is 16 ms smaller than non-musicians, and with a slower tempo (1000 ms IOI), musicians exhibited a 23 ms smaller standard deviation (Repp and Doggett, 2007). Here, three tempos were used, 350, 525, and 750 ms. Because the main effect of musical experience averaged over these tempos, it could be presumed that the standard deviations would be closest to those previously reported with a 500 ms IOI (i.e., 16 ms musicianship advantage). Indeed, the current results show musicians produced a standard deviation that is 15 ms smaller than non-musicians (Figure 2G). Together, the asynchrony and standard deviation results not only replicate research showing a musicianship advantage in sensorimotor synchronization, but do so with comparable magnitudes.

Circular statistics were also employed to assess our hypotheses. Whereas relative phase is related to asynchrony, vector length is related to standard deviation. Importantly, both of these metrics corroborated the linear statistics in showing that musicians yielded better sensorimotor synchronization performance than non-musicians in terms of smaller relative phase (smaller asynchrony) and larger vector length (less variance). Yet, each of these metrics describe different aspects of the synchronization performance. To address this, a rhythm score was created by combining accuracy, asynchrony, and standard deviation. Although accuracy did not show significant differences between musicians and non-musicians (*p* = 0.088), the rhythm score was able to identify significant differences in performance based on musical experience.

It is common for research studies to recruit musicians as defined by participants with musical instrument experience. Presumably, this is because instrumentalists are often trained to specifically move their fingers with precise timing, which forms the basis for many sensorimotor synchronization studies. Here, we characterized musicians to be inclusive of instrumentalists, singers and dancers – in other words, regardless of their prior training in finger moving. As such, it is encouraging, though perhaps not surprising, that such large musicianship effects were observed. This is in line with prior research indicating that singing experience improves a fundamental aspect of synchronization ability that may be deployed across different effectors, such as between voice and fingers (Dalla Bella et al., 2015).

### Effects of Age

It was hypothesized that younger adults would perform better than older adults. Previous research has demonstrated that younger adults yield an absolute asynchrony that is approximately 10 ms smaller than older adults (Bangert and Balota, 2012). Here, we show younger adults exhibit an asynchrony that is 13 ms smaller than older adults, thereby replicating not only the effect of younger adults performing better than older adults, but again, doing so with a similar magnitude. Similarly, previous research has shown younger adults’ accuracy during a rhythm reproduction task is approximately 90%, whereas older adults’ accuracy was around 70% (Iannarilli et al., 2013). The current data demonstrated 91% accuracy in younger adults, but 85% accuracy in older adults – slightly higher than the previous report for older adults, but very comparable to younger adult accuracy. However, this small difference in the older adult accuracy is likely due to the different tasks employed. Indeed, age-related differences in sensorimotor synchronization are sensitive to multiple factors, including task complexity (Serrien et al., 2000; Krampe et al., 2005; Iannarilli et al., 2013), musical training (Iannarilli et al., 2013; Thompson et al., 2015), and how old the older adults are (Drewing et al., 2006; McAuley et al., 2006; Turgeon et al., 2011). Taking these factors into account may help explain contradictory research indicating comparable synchronization performance between younger and older adults (Williams and Greene, 1993; Vanneste et al., 2001). Nonetheless, the current results support the hypothesis that younger adults exhibit better sensorimotor synchronization performance, as indexed by larger accuracy, vector length, rhythm score, and smaller asynchrony and standard deviation.

### Effects of Task

It was hypothesized that the on-beat task would yield better synchronization performance than the continuation or off-beat tasks. Previous research has reported a wide range of mean asynchrony values for the on-beat (29 to −90 ms), continuation (25 to −45 ms), and off-beat (12 to −90 ms) tasks (Engström et al., 1996; Rao et al., 1997; Repp, 2002; Jantzen et al., 2004; Flach, 2005; Serrien, 2008; Turgeon and Wing, 2012). For comparison, when we convert our absolute asynchrony to mean asynchrony, our results are in line with those previous reports (on-beat: −56 ms; continuation: −51 ms; off-beat: −71 ms). Yet, many of these previous reports show small significant differences (or no significant difference) between tasks when mean asynchrony is used as a metric of performance, such that the difference between continuation and on-beat ranges between 10 ms (on-beat closer to no asynchrony) (Rao et al., 1997) and 7 ms (continuation closer to no asynchrony) (Jantzen et al., 2004), whereas the difference between off-beat and on-beat ranges between 20 ms (on-beat closer to no asynchrony) (Jantzen et al., 2004) and −6 ms (off-beat closer to no asynchrony) (Repp, 2002). Again, our data is in line with these differences between tasks when assessing mean asynchrony: continuation minus on-beat = 5 ms (continuation closer to no asynchrony), off-beat minus on-beat = −15 ms (on-beat closer to no asynchrony). However, using mean asynchrony is problematic when trying to interpret optimal performance as “no asynchrony,” because high and low variability around zero asynchrony will still average to zero asynchrony regardless of variability, thereby reducing interpretability. Also, comparing differences around zero, even if they are significantly different, may have no meaning when they are equidistant from zero (e.g., −10 and 10 ms) because taps that are within the shortest possible reaction time (about 150 ms) are considered anticipatory (Mates et al., 1992; Repp and Su, 2013). While metrics such as vector length and standard deviation can quantify variability, absolute asynchrony can account for variability around zero and create a meaningful metric that associates “no asynchrony” with optimal performance. This is why we report absolute asynchrony here, and this is likely why we observed significant differences between tasks when using absolute asynchrony (Figure 4G), which supports the hypothesis that the on-beat task yields the best performance.

Similar to mean asynchrony, previous research has reported a wide range of standard deviations for the on-beat (24 to 80 ms), continuation (27 to 70 ms), and off-beat (58 to 120 ms) tasks (Engström et al., 1996; Rao et al., 1997; Jantzen et al., 2004; Serrien, 2008; Turgeon and Wing, 2012). Again, data from the current study falls within these ranges (Figure 4H), except for continuation (90 ms), which is somewhat larger than the range reported here. Nonetheless, it is unlikely that this is an indication that this data point is an outlier. First, 90 ms is still smaller than previous reports from the off-beat task and second, the previous studies reported here focused on healthy young adults, whereas our variability is inflated by including musicians, non-musicians, young adults and older adults. More importantly, prior studies generally observe significantly less variability during the on-beat task compared to continuation and off-beat tasks (Engström et al., 1996; Rao et al., 1997; Serrien, 2008), which contributed to the hypothesis that performance would be best during the on-beat task. Not only did the absolute asynchrony metric support this hypothesis (as discussed above), but support also came from our measures of variability (i.e., standard deviation and vector length) as well as accuracy and the composite rhythm score.

While it is common for studies to compare on-beat and continuation tasks or on-beat and off-beat tasks, it is less common to see a comparison of all three – or more specifically, a comparison between continuation and off-beat. Due to this lack of data, we had no specific hypothesis about any potential differences between the continuation and off-beat tasks and it is unclear why the continuation task was easier than the off-beat task. In fact, the opposite could be hypothesized because during the continuation task, there are no stimuli to assist in error correction. Therefore, variability would accumulate over time and the probability of large asynchronies would increase (Hary and Moore, 1987; Vorberg and Wing, 1996). Yet, performance during the continuation task was better than during the off-beat task, which probably arises from several factors. First, continuation was only performed for four beats (or taps) before the stimuli re-emerged, thereby providing an opportunity for error correction before participants had to continue with the continuation task. Second, the off-beat task has a slower tempo threshold for performance compared to the on-beat task (Repp, 2005a, b, 2007), and presumably by extension, the continuation tasks. Given that the fastest tempo performed (350 ms IOI) was (not coincidentally) the theoretical limit for off-beat performance in young adult musicians (Repp, 2005b), it is likely that the sensitivity of the off-beat task to tempo helped drive performance lower than continuation performance.

### Effects of Stimulus

It was hypothesized that bimodal (audio-visual) stimuli would yield better synchronization performance than with unimodal (audio-only, visual-only) stimuli. This hypothesis was based on previous research reporting lower standard deviations with bimodal, compared to unimodal, stimuli. Although the magnitude of the difference between bimodal and unimodal stimuli varies between 2 ms (unimodal has smaller standard deviation) and −55 ms (bimodal has smaller standard deviation) (Elliott et al., 2010, 2011; Wing et al., 2010; Wright et al., 2014; Blais et al., 2015; Roy et al., 2017), prior results generally indicate performance is better with bimodal stimuli. Here, the difference between bimodal and unimodal stimuli was −6 ms (Figure 5H), in line with previous results.

Interestingly, the other metrics of performance (relative phase, vector length, accuracy, asynchrony, rhythm score) only provided partial support for the hypothesis, such that bimodal performance was greater than visual-only, but not statistically different from audio-only. Fortunately, this result is not without precedent. Studies assessing asynchrony typically show either bimodal is comparable to the best unimodal asynchrony or that bimodal is between the two unimodal asynchronies (Wing et al., 2010; Wright et al., 2014; Blais et al., 2015; Roy et al., 2017). Here, we show the former using absolute asynchrony (Figure 5G). However, we observe the latter when mean asynchronies are calculated, such that bimodal (audio-visual) asynchrony (−57 ms) is between audio-only (−50 ms) and visual-only (−73 ms) asynchronies. Therefore, similar to standard deviation, the asynchrony data replicates previous results.

Although the asynchrony and standard deviation results are seemingly discrepant, they have been accounted for by an optimal integration model based on maximum likelihood estimation (Beers et al., 1999; Ernst and Banks, 2002; Ernst and Bülthoff, 2004), which suggests multiple modalities are combined by weighting each modality according to its relative reliability. Importantly, this model predicts that the combination of sensory modalities will reduce the variance of the underlying sensory representation. Furthermore, it predicts a shift in the mean of the underlying distribution toward the more strongly weighted modality. Therefore, the current results support these model predictions based on the standard deviation and mean asynchrony.

### Effects of Tempo

It was hypothesized that the medium tempo would yield better synchronization performance than the fast or slow tempos. This hypothesis was drawn from previous research indicating that the adult preferred tempo is between 400 and 700 ms IOI (Fraisse, 1982; Moelants, 2002; McAuley et al., 2006), and that synchronization performance declines outside the preferred tempo (Parncutt, 1994; McAuley et al., 2006; Delevoye-Turrell et al., 2014; Zamm et al., 2018). Although the current results assessing the composite rhythm score supported this hypothesis, our other metrics did not – which may reflect the utility of different performance metrics. For example, in a prior study, performance showed a U-shaped relationship with tempo, such that mean asynchrony and variance was minimal at the preferred tempo (Delevoye-Turrell et al., 2014). When we calculate mean asynchrony, we begin to see a similar effect as previously reported such that slow, medium, and fast tempos yielded −59, −56, and −65 ms asynchronies, respectively, although only the medium and fast tempos differ significantly (*p* = 0.001). Yet, no such relationship was observed when we calculate variance. Focusing on a different metric, a recent study has shown that the coefficient of variation (CV = standard deviation/mean) indexes optimal performance at the preferred tempo (Zamm et al., 2018), but we did not observe this (CV slow = 0.66, CV medium = 0.66, CV fast = 0.69). In fact, our CV measures were more closely in line with previous data indicating that CV is relatively consistent across tempos between 300 and 1200 ms IOI (McAuley et al., 2006). Nonetheless, our data would suggest that no single metric fully captures synchronization performance, as trade-offs may occur. Specifically, asynchrony and standard deviation were largest (worst) during the slow tempo, and yet, accuracy was the greatest. Performance during the fast tempo showed the opposite relationship, while performance during the medium tempo was close to the best asynchrony, standard deviation and accuracy – but never the best in any single metric. As such, the rhythm score captures these tradeoffs between metrics to show that synchronization performance, as a whole, is best during the medium tempo.

Overall, the results suggest the data is both valid (via replication) and reliable (via ICC), and so an exploratory analysis was conducted on the rhythm score, which exhibited an interaction between Tempo × Age × Task × Experience. Some of this complex interaction arises from two cases where off-beat performance was greater than continuation and comparable to on-beat performance: young musicians during the slow tempo (Figure 8A) and older non-musicians during the fast tempo (Figure 8C). Regarding the former, young adult musicians are known to have a faster tempo threshold for off-beat performance (Repp, 2005a, b, 2007), which may help account for their ability to perform the off-beat task so well. Additionally, when conducting the off-beat tasks during the slow tempo, participants must sub-divide the rhythm so that the time between taps and stimuli is 375 ms. This is interesting because only the young adult musicians were able to conduct the on-beat and continuation tasks relatively well at the fast (350 ms) tempo. As for why young musicians’ off-beat performance was better than continuation during the slow tempo, this likely stems from the slow tempo allowing variability to accumulate over time during continuation performance, leading to larger asynchronies (Hary and Moore, 1987; Vorberg and Wing, 1996). Indeed, when less time is allowed for variability to accumulate (i.e., medium tempo), continuation performance appears to be improved and that visual inspection of Figure 8 shows greater continuation performance during the medium, compared to slow, tempo for each group (young/older musicians/non-musicians). On the other hand, older adult non-musicians during the fast tempo also exhibited better off-beat task performance compared to continuation. It is speculated that because fast IOIs during off-beat tasks is known to cause an unintentional phase shift in performance to on-beat (Haken et al., 1985; Wimmers et al., 1992), it is possible that older adult non-musicians were drawn to tapping on-beat. And because older adult non-musicians yielded the largest asynchronies, producing a large late asynchrony on-beat would yield a close early asynchrony off-beat, resulting in improved performance. Regardless, our interpretation of the observed four-way interaction is speculative and not exhaustive because it was exploratory. Furthermore, the effect was not very strong and would benefit from replication. We hope that by highlighting this interaction, other researchers will be able to form experiments specifically designed to assess the complex interactions between age, experience, tempo, task, and stimulus type.

### Limitations

Although the current results support the notion that mobile tablets can be used for research data collection assessing synchronization abilities, there are several limitations to the technology and to the study that should be addressed. In terms of the technology, mobile devices are not as powerful as the standard desktop computer used in most research paradigms. The limitations of these devices are well-established (Ng and Dietz, 2014; Ritter et al., 2015; Arsintescu et al., 2017; Begel et al., 2017), and of particular concern is (1) the variability in producing stimuli when requested and (2) the latency of registering touchscreen input. Here, we measured the standard deviation of the IOI to be 14 ms for auditory stimuli and 13 ms for visual stimuli. Although this is not ideal, our data indicates that it was precise enough to replicate previous results from standard laboratory experiments and to yield excellent test–retest reliability.

As for latency of the touchscreen input, we relied on a previously published assessment for the tablet we used in the study, a Microsoft Surface Pro 3 (Deber et al., 2016). In that study, input lag was measured to be 69.53 ms on 354 out of 424 trials. However, on 70 out of 424 trials, the lag was one screen refresh longer. On the Microsoft Surface Pro 3 for the current study, the refresh rate was set to 60 Hz. Therefore, to correct for input lag, all recorded tap data were adjusted by the weighted average of 72.28 ms. To assess the impact of not correcting for input lag, we re-analyzed the current data without a lag correction. As expected, relative phase no longer showed a negative mean asymmetry, but rather, was late or closer to zero asynchrony. To a much lesser extent, absolute asynchrony was also affected. Importantly, statistical comparisons between and within groups were largely unchanged, supporting the notion that research with mobile devices can be conducted without lag correction as long as the research question is not reliant on absolute timing information.

It is important to note that input lag is device specific. Therefore, studies that do not account for the input lag will likely report response times longer than what would be obtained from typical research equipment. Fortunately, this is not necessarily a problem if all participants are using the same type of device (make, model, and OS) because everyone will have similar lag – assuming comparable CPU and memory is available as needed for the application. When different devices are used, it would be necessary to either collect device information and correct for known input lag, or design an experiment that relies solely on within-subject comparisons that are not based on absolute timing.

Aside from technological limitations, several study design limitations should be noted. First, the number of participants was limited, particularly in the older adults group. This restriction was a product of the number of participants were able to recruit in a set period of time. Fortunately, an assessment of the 95% confidence intervals indicates a relatively narrow range around the mean difference between age groups (e.g., rhythm score mean difference = 0.52, CI = 0.32–0.71) and the magnitude of age differences is in line with previous research. Second, it should be noted that our assessment of “rhythm abilities” was limited to factors that were tested and that there are many other facets to rhythm abilities not tested here. Finally, the sensorimotor synchronization assessment did not randomize trials. As such, a practice based bias may exist that places the slow tempos (conducted first) at a disadvantage, and the fast tempos (conducted last) at an advantage. However, all participants were given enough practice at the medium tempo to feel comfortable with the tasks, which should have helped offset practice effects. Regardless, future iterations of the sensorimotor synchronization assessment will include a randomization procedure.

Related to the lack of randomization, the sensorimotor synchronization assessment does not offer the flexibility to easily manipulate parameters of the game or even use it on a platform other than the Microsoft Surface Pro 3. Such changes would require programing knowledge in Unity and possibly C# (depending on requirements), which may limit the utility of this particular assessment for others. As such, we are currently developing a new version for iOS that offers more flexibility such as randomization and the ability to select particular tasks, tempos, and stimulus types. Researchers interested in using either of these versions should contact us. Nonetheless, the point of this research was to show that consumer devices using game engine software can yield data that is on-par with typical lab-based research. The creation of lab-based paradigms often requires some form of programing knowledge such as in E-prime (E-Basic/Visual Basic), Psychophysics Toolbox (Matlab), Presentation (SDL/PCL) or PsychoPy (Python) to name a few. Therefore, it is reasonable to suggest that some researchers may benefit from learning Unity (C#) and using it for experimental task presentation – depending on the research goals.

## Conclusion

The results of this research suggest that tablet-based mobile platforms presenting software generated on consumer game engines can be a valid (via replication) and reliable (via ICC) means to collect measures of sensorimotor synchronization ability. While it is recognized that different mobile platforms offer different performance abilities, the current research provides promising evidence that at-home based research may benefit from the widespread use of consumer-based mobile electronics, even when the research paradigm demands relatively precise timing. Furthermore, data from the sensorimotor synchronization assessment provided interesting preliminary evidence for a complex relationship between age, musical experience, tempo, and task. Future research will be needed to better understand the parametric relationship between these factors, which will shed important light on how sensorimotor synchronization ability changes across the lifespan and the potential for musical training to remediate or avoid age-related declines.

## Ethics Statement

This study was carried out in accordance with the recommendations of the UCSF IRB with written informed consent from all subjects. All subjects gave written informed consent in accordance with the Declaration of Helsinki. The protocol was approved by the Committee for Human Research at the University of California, San Francisco.

## Author Contributions

TZ, NP, AN, and AG designed the experiments. NP and AN collected the data. TZ analyzed the data. TZ, NP, and AG prepared the manuscript.

## Conflict of Interest

The authors declare that the research was conducted in the absence of any commercial or financial relationships that could be construed as a potential conflict of interest.
